# Supplementary material for: Effect of digitizing Community Health Information System on the delivery and use of maternal and child health services: Propensity score matching analysis
Source: PLoS One. 2026 May 28;21(5):e0350270. doi: 10.1371/journal.pone.0350270 (PMC13218477; doi:10.1371/journal.pone.0350270)
Supplement: Attachment — Submitted filename: Point-by-point response.docx [file pone.0350270.s003.docx]

**A point-by-point response to reviewers and editor**

PONE-D-25-50830: Effect of digitizing Community Health Information System on the delivery and use of maternal and child health services: Propensity score matching analysis

Dear Editor,

We, the authors, would like to thank the reviewer for their valuable comments. Our point-by-point responses to the reviewer and editor are below each comment. We also reviewed to ensure that this manuscript version conforms to the journal style.

Journal requirements:

When submitting your revision, we need you to address these additional requirements.

1. Please ensure that your manuscript meets PLOS ONE's style requirements, including those for file naming. The PLOS ONE style templates can be found at

<https://journals.plos.org/plosone/s/file?id=wjVg/PLOSOne_formatting_sample_main_body.pdf> and

<https://journals.plos.org/plosone/s/file?id=ba62/PLOSOne_formatting_sample_title_authors_affiliations.pdf>

2. Thank you for stating the following in the Acknowledgments Section of your manuscript:

“The SSD and eCHIS projects are funded by the BMGF and CIFF, respectively, and implemented through JSI. The funders have no role in the interpretation and implications of the content in this paper. This responsibility lies solely with the authors.”

We note that you have provided funding information that is currently declared in your Funding Statement. However, funding information should not appear in the Acknowledgments section or other areas of your manuscript. We will only publish funding information present in the Funding Statement section of the online submission form.

Response: We have removed all funding-related statements from the Acknowledgments section in accordance with journal requirements. The revised Acknowledgments now read:

*“We extend our gratitude to the Ministry of Health, woreda health offices, health center staff, and HEWs at the eCHIS project implementation sites for their essential engagement. We acknowledge the interviewers and supervisors for their hard work, dedication, and timely completion of the fieldwork. Finally, we are deeply grateful to all study participants who took the time to respond to the survey questionnaires and provided invaluable information.”*

Please remove any funding-related text from the manuscript and let us know how you would like to update your Funding Statement. Currently, your Funding Statement reads as follows:

“The SSD and eCHIS projects are funded by the BMGF and CIFF, respectively, and implemented through JSI. The funders have no role in the interpretation and implications of the content in this paper. This responsibility lies solely with the authors.”

Response: We confirm that the Funding Statement remains unchanged and is included only in the designated section of the submission form:

*“The SSD and eCHIS projects are funded by the Gates Foundation and the CIFF, respectively, and implemented through JSI. The funders had no role in the interpretation of the findings or the conclusions presented in this manuscript. The authors bear sole responsibility for the content.”*

Please include your amended statements within your cover letter; we will change the online submission form on your behalf.

Response: The amended statements are included in the cover letter.

Please review your reference list to ensure that it is complete and correct. If you have cited papers that have been retracted, please include the rationale for doing so in the manuscript text, or remove these references and replace them with relevant current references. Any changes to the reference list should be mentioned in the rebuttal letter that accompanies your revised manuscript. If you need to cite a retracted article, indicate the article’s retracted status in the References list and also include a citation and full reference for the retraction notice.
Response: We have carefully reviewed and updated the reference list to ensure completeness, accuracy, and consistency with journal guidelines. No retracted articles were cited. Any minor corrections have been incorporated and are reflected in the revised manuscript.

**Reviewers' comments:**

Review Comments to the Author
Reviewer #1: Background:
Ensure all claims are properly referenced. For example, line 79 mentions.
Response: Thank you. We have reviewed the Background section and added appropriate references to support all key claims.

Methods:
Highlight the imbalance and provide explanation whether weighting was applied during the analysis

Response: Thank you for this important point. We have clarified this in the Discussion and Limitations sections. Specifically, we now note that residual imbalance may persist due to the study design and that matching could not fully address differences between groups. The revised text reads:

*“Additionally, because the study was not originally designed to evaluate eCHIS, baseline imbalances between intervention and comparison groups may persist despite matching. These likely reflect differences in geographic, socioeconomic, and health system contexts across clusters, which could influence both exposure and outcomes. Residual confounding may therefore affect the estimated effects.”* ***Page 19, lines 323-27 of the revised manuscript with track changes***

Ensure consistency in describing age ranges: women of reproductive age (15–49 years)” should be explicitly linked to MNH and family planning data collection.

Response: Thanks for the feedback. This has been revised for clarity and consistency. The text now reads:

*“The SSD baseline survey used a stratified, multistage sampling approach to recruit three groups of women aged 15–49 years: 3,611 women of reproductive age, 1,922 women with infants aged 0–11 months, and 1,368 women with children aged 12–23 months.”* ***Page 7, lines 115-17 of the revised manuscript with track changes***

Ensure S1 File is properly cited and that the text indicates what S1 File contains

Response: Noted. The S1 File is now properly cited in the text, and its contents are clearly described.

Measurement:
Use consistent past tense for methods

Response: Thank you. We have revised the Methods section to ensure consistent use of past tense throughout. ***Page 9, line 149 of the revised manuscript with track changes***

Table 1: Consider clarifying in text that the denominator differs per indicator and link this explicitly to survey design.

Response: We have clarified this in the manuscript. The revised text specifies that:

*“The dependent variable of interest was the RMNCH service uptake, which was expected to be affected by the eCHIS intervention and was measured by the household surveys among three groups of women aged 15–49 years: women of reproductive age for family planning indicators, women with infants aged 0–11 months for MNH indicators, and women with children aged 12–23 months for immunization indicators.”* ***Page 8-9, line 143-46 of the revised manuscript with track changes***

Explain how the first component was used to rank households
Response: We have clarified this as follows:

*“The first principal component, which captured the largest variation in asset ownership and living conditions and had the highest eigenvalue, was retained to represent underlying socioeconomic status and used to generate a continuous wealth score for each household.”* ***Page 10, line 159-62 of the revised manuscript with track changes***

Add a short justification for why two out of three criteria was chosen, possibly referencing a previous study or standard.

Response: Thank you for this important question. The decision to classify women as having autonomy when they met at least two of the three criteria was made to ensure that the measure reflects a minimum consistent level of decision-making power across multiple domains, rather than isolated or context-specific participation in a single domain. Using a two-out-of-three threshold reduces the likelihood of misclassifying women as autonomous based on a single favorable response and provides a more robust proxy of sustained autonomy in household decision-making. This approach is consistent with practices in demographic and health research where composite empowerment indicators are constructed to capture multidimensional constructs rather than single-item responses.

Analysis:
Clarify why kebele-level PS were used, not individual-level, to help readers understand clustering at community level.
Response: We have clarified this in the Methods section:

*“Propensity scores were calculated for each kebele to estimate adjusted intervention effects, using a logit model for the three groups of mothers separately, accounting for the clustered sampling design in which households within the same kebele share similar contextual characteristics and reducing bias from correlated exposures.”* ***Page 11, lines 172-75 of the revised manuscript with track changes***

Stepwise-backward selection: mention that this was done separately for each subgroup, which you do, but could be clearer in flow.

Response: Thank you. We have clarified that this procedure was conducted separately for each subgroup to improve readability and transparency.

Include a flowchart of sampling and survey process.
Response: We appreciate the suggestion. A flowchart illustrating the sampling and survey process has been added as Figure 1. ***Page 7, lines 131 of the revised manuscript with track changes***

Discussion:
Several points are repeated: eCHIS impact on tracking, defaulter tracing, and workflow efficiency appears multiple times (lines 240–256, lines 257–266).
Response: Thank you. The Discussion has been revised to remove redundancies and streamline overlapping content.

References to mHealth interventions improving maternal and child health outcomes (lines 263–266) partially overlap with earlier discussion (lines 257–262)
Response: We have consolidated overlapping references to mHealth evidence into a single coherent paragraph to improve clarity.

Sentences (lines 279-289) could be split for an easier read
Response: These sentences have been revised and split to improve readability.

While the discussion references prior studies, it could emphasize which results are consistent vs. which are novel in the Ethiopian context.
Response: We have strengthened the Discussion to explicitly distinguish findings consistent with prior literature from those that are novel in the Ethiopian context.

Some sentences describe improvements, but it would be stronger to directly link each improved outcome to the eCHIS feature responsible

Response: Thank you for these constructive comments. We have comprehensively revised the Discussion section to improve clarity, coherence, and analytical depth. Specifically, we removed repetitive statements and consolidated overlapping discussions of mHealth evidence into a single coherent paragraph. In addition, we strengthened the Discussion by clearly distinguishing findings that are consistent with existing literature from those that are novel to the Ethiopian context. ***Page 16-19, lines 257-31 of the revised manuscript with track changes***

Conclusion:
some sentences are redundant (lines 291–294 vs. 295–298). Condensing these would make the section more concise.
Response: Thank you. The Conclusion has been condensed to remove repetition and improve clarity. ***Page 20, lines 335-37 of the revised manuscript with track changes***

What type of investment or next steps are more critical and why?
Response: We have expanded this section as follows:

*“However, maximizing its impact will require addressing implementation bottlenecks, strengthening infrastructure, and aligning with service delivery policies.”* ***Page 20, lines 310-11 of the revised manuscript with track changes***

Future research focus could be more precise.

Response: We have refined this to state:

“Further research is required to **examine implementation bottlenecks of eCHIS to inform context-specific scale-up strategies** and assess its showcase eCHIS’s cost-effectiveness and long-term impacts in transforming the HEP to improve primary health care outcomes, efficiency, and service quality.” ***Page 20, lines 341-42 of the revised manuscript with track changes***

Reviewer #2: the research article titled "Effect of digitizing Community Health Information System on the delivery and use of
"Maternal and child health services: Propensity score matching analysis" is a very interesting and urgent issue in today's world. And hence, it fulfills the minimum standard for publication in the PLOS ONE journal. All the statistical analyses are very sound, and the findings revealed the current situations regarding maternal and child health. And finally, the conclusion and the findings are constructive enough.

Response: We sincerely thank the reviewer for the positive and encouraging feedback. We appreciate the recognition of the study’s relevance, methodological rigor, and contribution to understanding maternal and child health service delivery.
